# Biphasic regulation of tumorigenesis by PTK7 expression level in esophageal squamous cell carcinoma

**DOI:** 10.1038/s41598-018-26957-6

**Published:** 2018-06-04

**Authors:** Won-Sik Shin, Jungsoo Gim, Sungho Won, Seung-Taek Lee

**Affiliations:** 1 Department of Biochemistry, College of Life Science and Biotechnology, Yonsei University, Seoul, Republic of Korea; 2 Department of Biomedical Science, College of Natural Science, Chosun University, Gwangju, Republic of Korea; 3 Graduate School of Public Health, Seoul National University, Seoul, Republic of Korea; 4 Interdisciplinary Program for Bioinformatics, Seoul National University, Seoul, Republic of Korea

## Abstract

Protein tyrosine kinase 7 (PTK7), also known as colon carcinoma kinase 4 (CCK-4), is a member of the catalytically defective receptor protein tyrosine kinase family and is upregulated in various cancers, where it is known to act as either an oncoprotein or a tumor suppressor. To understand the contrasting roles of PTK7 in tumorigenesis, we analyzed the tumorigenic characteristics of esophageal squamous cell carcinoma (ESCC) cells with low levels of endogenous PTK7 expression (TE-5 and TE-14 cells) and high levels of expression (TE-6 and TE-10 cells) after transfections with a PTK7 expression vector. PTK7 overexpression increased the proliferation of TE-5 and TE-14 cells but decreased the proliferation of TE-6 and TE-10 cells. In the ESCC cells, proliferation, migration, and invasion were initially increased and then decreased according to PTK7 expression levels, which were mirrored by initial increases and then decreases in the tyrosine phosphorylation of cellular proteins and phosphorylation of Src, Akt, and ERK. In ESCC patients included in The Cancer Genome Atlas database, those with higher PTK7 mRNA levels had a longer overall survival and lower relative risk than those with lower PTK7 mRNA levels. These results demonstrate that PTK7 biphasically regulates tumorigenesis in ESCC.

## Introduction

Esophageal cancer is the eighth most common cancer and the sixth most common cause of death from cancer worldwide^1^. The most frequent types of esophageal cancer are esophageal adenocarcinoma (EAC) and esophageal squamous cell carcinoma (ESCC), with high incidences in Western and Asian countries, respectively^2^. Despite the advances in multidisciplinary treatment strategies and diagnostic imaging modalities, only 15 to 25% of esophageal cancer patients survive five years after their diagnosis^3^. Thus, the identification of biomarkers and the understanding of molecular mechanisms of esophageal cancer are important to improve patients’ prognoses.

One protein found to be upregulated in ESCC^4^ and other cancers, such as colorectal cancer^5–7^, is protein tyrosine kinase 7 (PTK7). PTK7 comprises an extracellular domain with seven immunoglobulin-like domains, a transmembrane domain, and a catalytically defective tyrosine kinase domain^8–10^, and thus is a member of pseudokinases^11^. PTK7 is involved in planar cell polarity (PCP) and canonical and non-canonical Wnt signaling during development^12^ via interactions with a non-canonical Wnt/PCP ligand, Wnt5A^13^, Wnt receptors such as Fz7 and LRP6^14,15^, and intracellular Wnt signaling proteins such as Dvl and β-catenin^16,17^. The functions of PTK7 are vital, as PTK7-deficient mice die perinatally with defects in neural tube closure and stereociliary bundle orientation similar to those observed in PCP mutant mice and frogs^18^.

There are several mechanisms by which PTK7 may influence cancer^19–21^. For example, PTK7 increases the proliferation, survival, migration, and invasion of cells and wound healing by activating ERK, JNK, p38, and NF-κB signaling pathways, whereas it decreases apoptosis by suppressing the activation of caspase-9 and -10^4,19–24^. PTK7 also increases angiogenesis through activation of KDR^25^. In contrast, PTK7 may also function as a tumor suppressor, as it is downregulated in other cancers such as melanoma and clear cell renal cell carcinoma^26,27^. In addition, a decrease in PTK7 expression is associated with a poor prognosis in patients with epithelial ovarian carcinoma^28^. In lung squamous cell carcinoma cells, the overexpression of PTK7 decreases cell proliferation, migration, and invasion by suppressing ERK and Akt phosphorylation^29^.

To clarify the role of PTK7 in ESCC tumorigenesis, we compared PTK7 expression levels with physiologic changes in these cells. We also analyzed the relationship between *PTK7* mRNA levels and the survival of ESCC patients using transcriptome sequencing (RNA-seq) data from The Cancer Genome Atlas (TCGA) database.

## Results

### *PTK7* is expressed at high levels in numerous cancers

**Figure 1:**
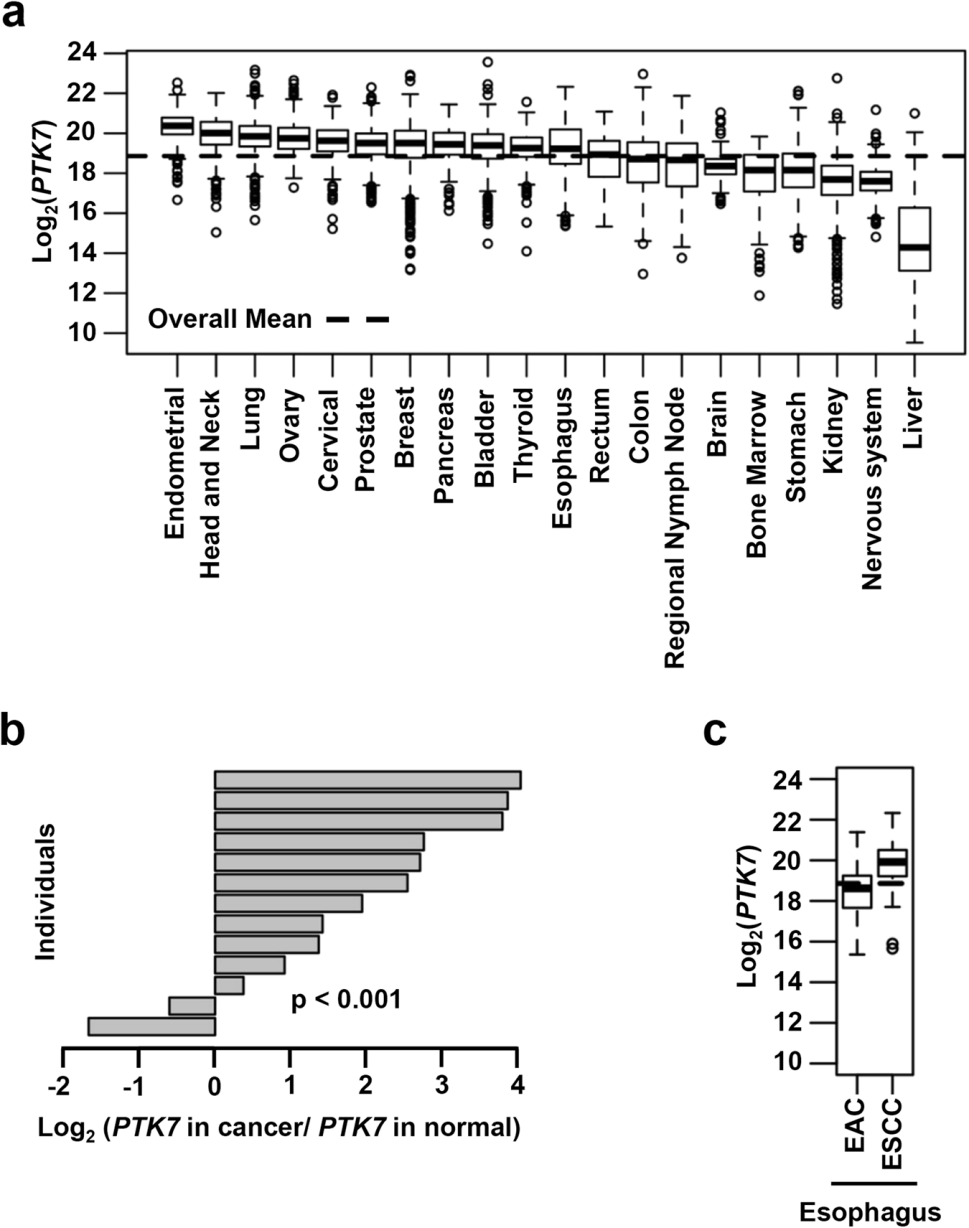
*PTK7* mRNA levels in various cancer types. (**a**,**c**) *PTK7* mRNA levels in tissue samples from 20 cancer types, including esophageal cancer (**a**) and in samples defined as EAC and ESCC (*n = *96 each) (**c**), included in the database from TCGA. The dotted horizontal lines indicate the overall mean level from all cancer types. (**b**) Ratios of *PTK7* mRNA levels in esophageal tumor samples to those in matched adjacent normal tissue samples in 13 sample pairs.

We analyzed RNA-seq data from TCGA to assess *PTK7* mRNA levels in various cancer tissues. In samples of endometrial, head and neck, lung, ovary, cervical, prostate, breast, pancreatic, bladder, thyroid, and esophageal cancers, the mean *PTK7* mRNA levels were higher than the overall mean level from all cancer tissues (Fig. 1a). Thirteen pairs of esophageal cancer and matched normal tissue samples were found in the database. A paired analysis showed that the *PTK7* mRNA level was higher in esophageal cancer tissues than in adjacent normal tissues (*P* < 0.001) (Fig. 1b). Among the 198 esophageal cancer samples in the database from TCGA, we extracted 96 EAC and 96 ESCC samples; six samples were undefined. The *PTK7* mRNA level was higher in ESCC samples than in EAC samples (Fig. 1c). These data demonstrate that in most cancer types, including esophageal cancer, PTK7 expression is upregulated.

### PTK7 overexpression increases the proliferation of PTK7-low ESCC cells but decreases the proliferation of PTK7-high ESCC cells

**Figure 2:**
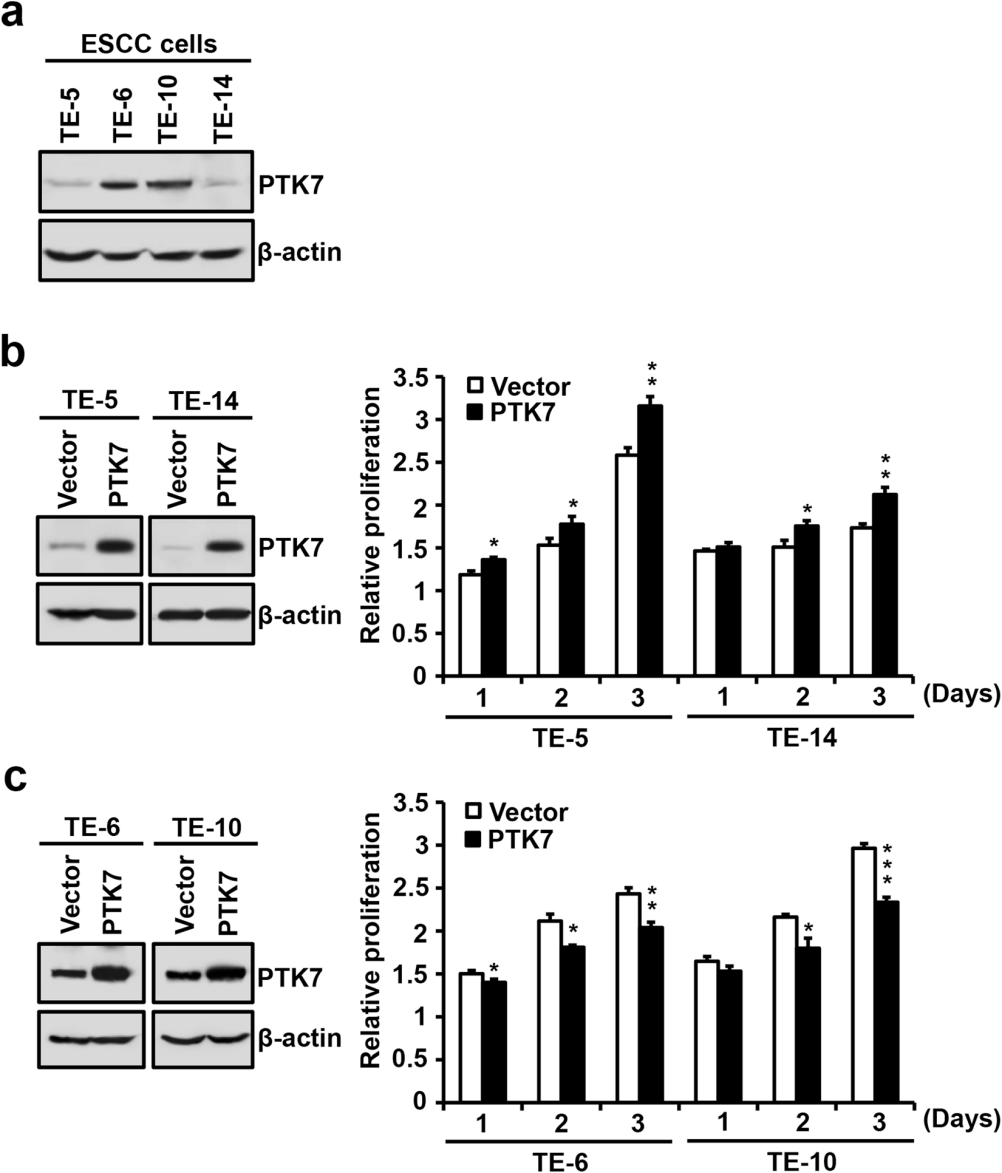
Effect of PTK7 overexpression on the proliferation of ESCC cells. Representative Western blots showing endogenous expression levels of PTK7 in ESCC TE-5, TE-6, TE-10, and TE-14 cells (**a**). PTK7-low TE-5 and TE-14 cells (**b**) and PTK7-high TE-6 and TE-10 cells (**c**) were transfected with a PTK7 expression vector (pcDNA3-PTK7-FLAG; PTK7) or an empty vector (pcDNA3; Vector) as the control. Twenty-four h later, Western blotting and proliferation assays were performed. Each bar represents the mean ± standard deviation from three independent experiments. *P < 0.05, **P < 0.01, ***P < 0.001 *vs*. vector control. Samples derived from the same experiment and gels/blots were processed in parallel. The blots were cropped to focus upon the specific proteins indicated. Uncropped images of blots are shown in Supplementary Fig. S4.

To examine the oncogenic role of PTK7, we ectopically expressed it in two ESCC cell lines with low endogenous levels of expression (TE-5 and TE-14) and two cell lines that highly express PTK7 (TE-6 and TE-10) (Fig. 2a). Ectopic expression of PTK7 increased the proliferation of TE-5 and TE-14 cells (i.e., PTK7-low ESCC cells) (Fig. 2b), whereas the proliferation of the PTK7-high TE-6 and TE-10 cells decreased with ectopic expression (Fig. 2c). This result was unexpected, as previous evidence showing PTK7 knockdown decreased the proliferation of TE-10 and TE-11 cells indicated that PTK7 is an oncogene in ESCC^4^.

### Biphasic oncogenic regulation of ESCC cells by PTK7 expression

**Figure 3:**
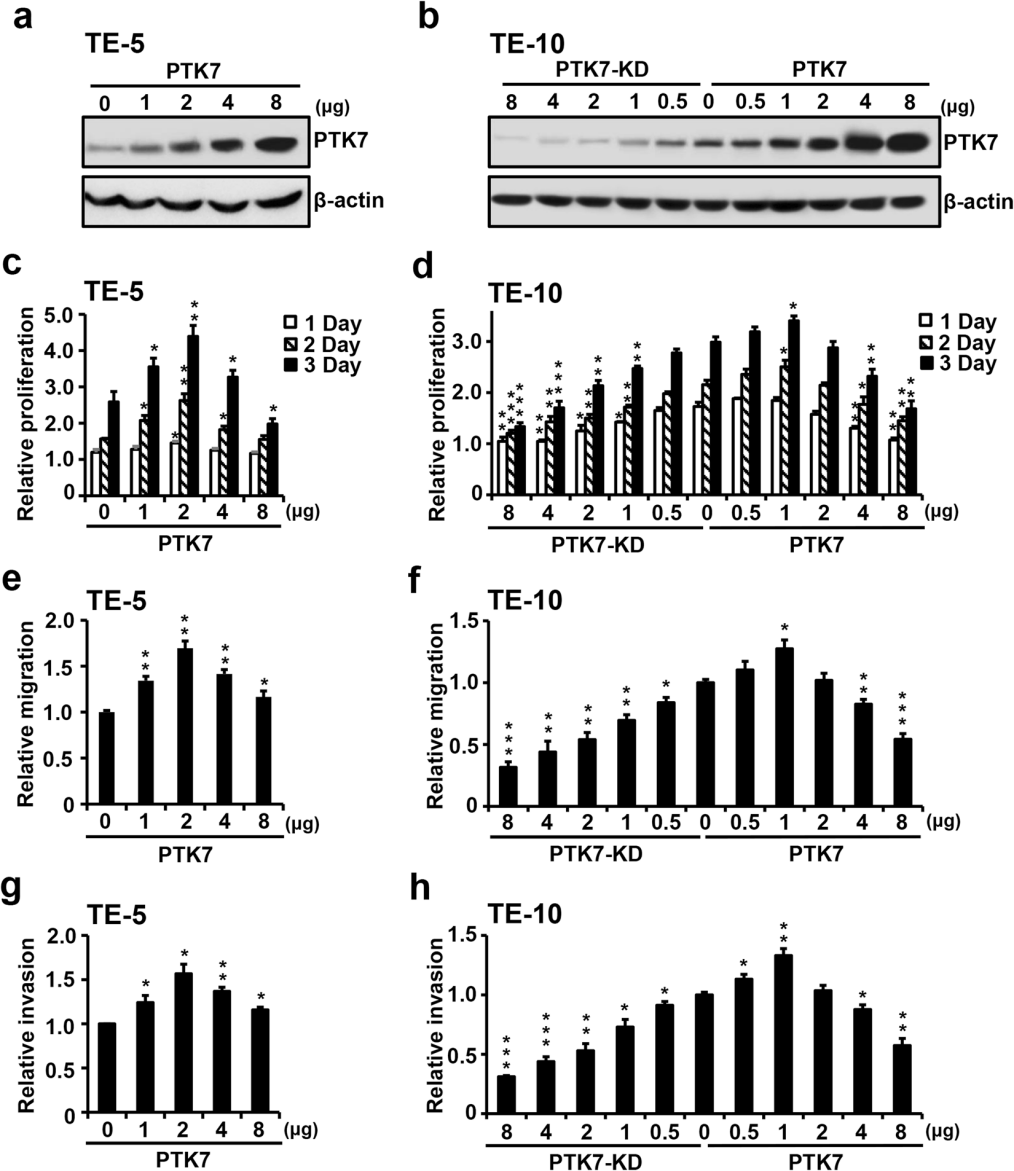
Biphasic oncogenic regulation of ESCC TE-5 and TE-10 cells by PTK7 expression. Representative Western blots showing PTK7 levels in PTK7-low TE-5 cells 24 h after transfection with increasing amounts of the PTK7 expression vector (pcDNA3-PTK7-FLAG; PTK7) (**a**), and in PTK7-high TE-10 cells transfected with various amounts of a PTK7 knockdown vector (pLKO.1-shRNA-PTK7-6434; PTK7-KD) or the PTK7 expression vector (**b**). Proliferation of the TE-5 (**c**) and TE-10 (**d**) cells was analyzed for 3 days. Migration and invasion of the TE-5 (**e** and **g**) and TE-10 (**f** and **h**) cells were analyzed 24 h after transfection. Each bar represents the mean ± standard deviation from three independent experiments. *P < 0.05, **P < 0.01, ***P < 0.001 *vs*. day 0 (**c** and **d**) or 0 μg of PTK7 expression vector (**e** to **h**). Samples derived from the same experiment and gels/blots were processed in parallel. The blots were cropped to focus upon the specific proteins indicated. Uncropped images of blots are shown in Supplementary Fig. S4.

To further examine the unexpected findings regarding PTK7 expression levels and ESCC cell proliferation, we next analyzed the oncogenic properties of ESCC cells expressing low to high levels of PTK7. Increasing amounts of the PTK7 expression vector were transfected into PTK7-low TE-5 and TE-14 cells (Fig. 3a and Supplementary Fig. S1a), and increasing amounts of the expression or a knockdown vector were transfected into PTK7-high TE-10 and TE-6 cells (Fig. 3b and Supplementary Fig. S1b). Inverted U-shaped dose-effect curves were observed with regard to the effects of PTK7 expression on cell proliferation (Fig. 3c and d and Supplementary Fig. S1c and d), migration (Fig. 3e and f and Supplementary Fig. S1e and f), and invasion (Fig. 3g and h and Supplementary Fig. S1g and h) of the ESCC cells, with increases from low levels promoting these oncogenic properties and higher levels suppressing them. The maximal oncogenic effects were found in PTK7-low TE-5 and TE-14 cells transfected with 2 μg or PTK7-high TE-10 and TE-6 cells transfected with 1 μg of the PTK7 expression vector. These results show that increasing levels of PTK7 expression biphasically regulate tumorigenesis, with an enhancement of oncogenic properties in ESCC cells at an optimal level of expression.

### Biphasic regulation of signaling protein phosphorylation by PTK7 expression

**Figure 4:**
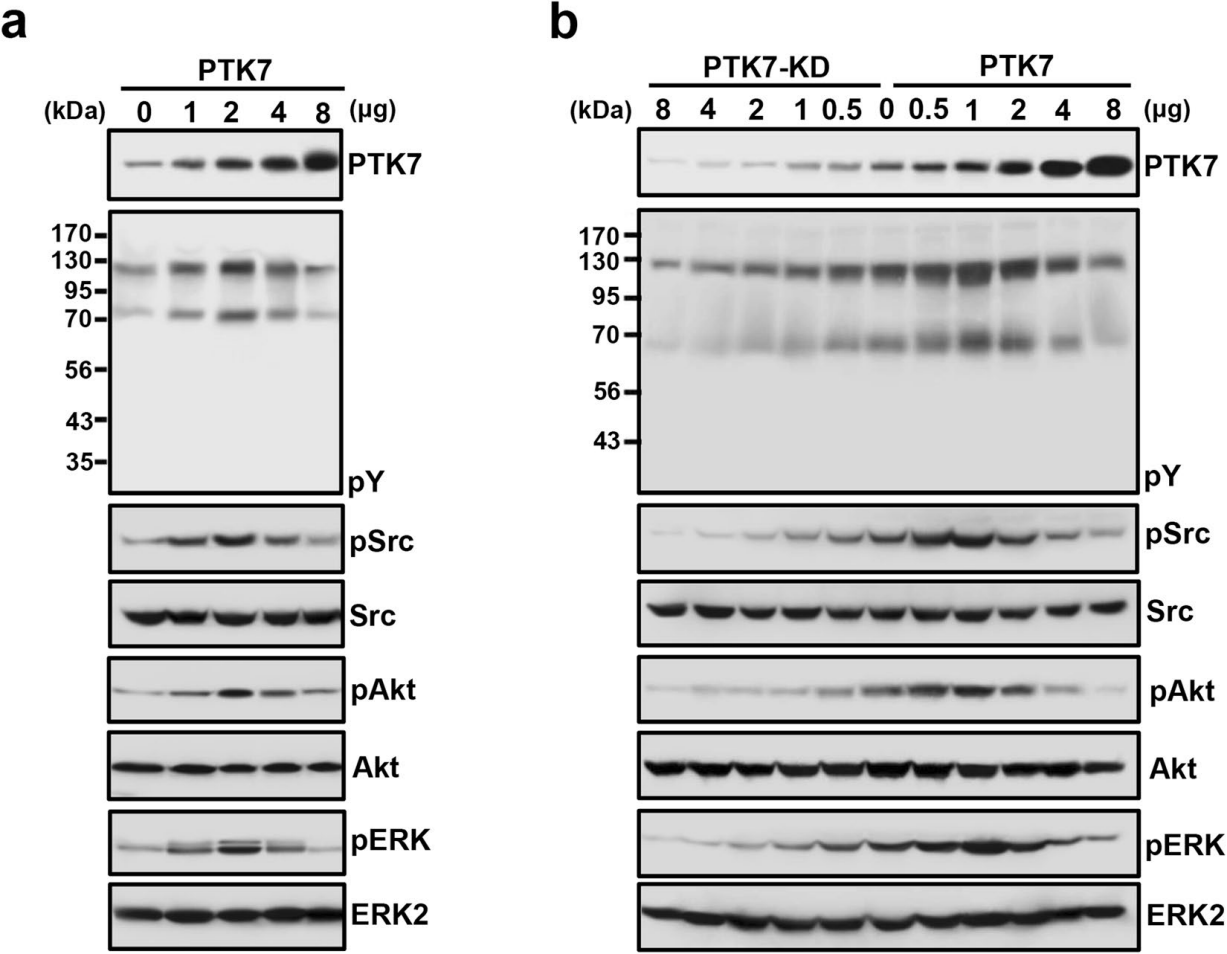
Biphasic regulation of protein phosphorylation in ESCC TE-5 and TE-10 cells by PTK7 expression. Representative Western blots from PTK7-low TE-5 cells 48 h after transfections with increasing amounts of the PTK7 expression vector (pcDNA3-PTK7-FLAG; PTK7) (**a**), and PTK7-high TE-10 cells transfected with various amounts of a PTK7 knockdown vector (pLKO.1-shRNA-PTK7-6434; PTK7-KD) or the PTK7 expression vector (**b**). Levels of tyrosine-phosphorylated cellular proteins (pY), as well as phosphorylated Src, Akt, and ERK, are shown. Numbers to the left of the blots indicate the molecular mass of the marker proteins (kDa). Samples derived from the same experiment and gels/blots were processed in parallel. The blots were cropped to focus upon the specific proteins indicated. Uncropped images of blots are shown in Supplementary Fig. S4.

We next investigated whether PTK7 expression biphasically regulates the phosphorylation of signaling proteins in ESCC cells. We found that, in accordance with the tumorigenic effects, increasing levels of PTK7 biphasically regulated the tyrosine phosphorylation of cellular proteins as well as the phosphorylation of Src, Akt, and ERK, with maximal phosphorylation achieved with 2 μg of the PTK7 expression vector in PTK7-low TE-5 and TE-14 cells (Fig. 4a and Supplementary Fig. S2a) or 1 μg of that in PTK7-high TE-10 and TE-6 cells (Fig. 4b and Supplementary Fig. S2b).

### High PTK7 expression in ESCC is associated with increased patient survival

**Figure 5:**
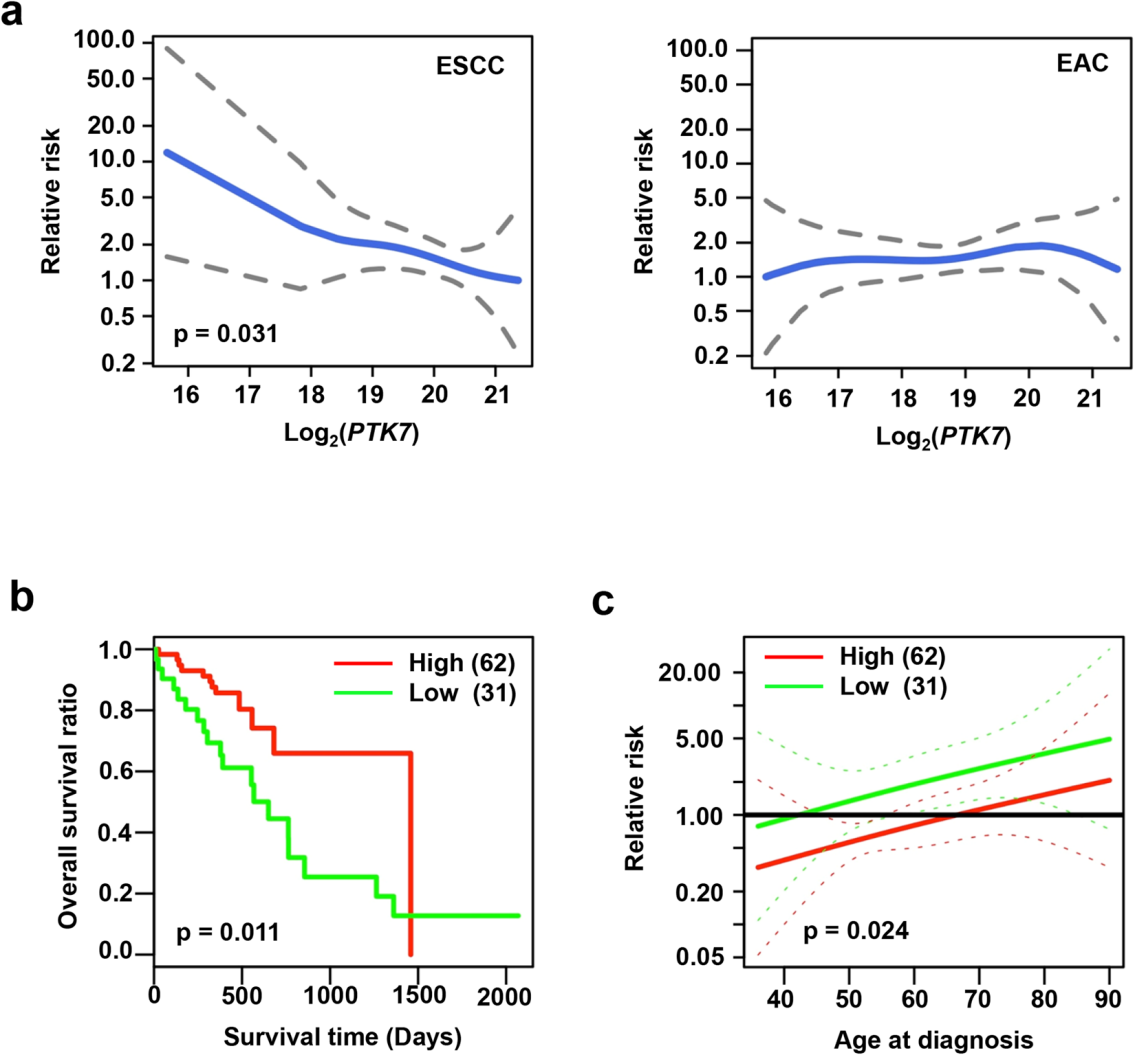
Risk and survival analyses of ESCC patients according to PTK7 expression. (**a**) Cox proportional hazards models with spline terms of PTK7 expression in patients with ESCC (left) and EAC (right) to analyze the relationship between relative risk and *PTK7* mRNA level. Kaplan–Meier curve for log-rank analysis of overall survival (**b**) and Cox proportional hazards model for analysis of relative risk at a specific age at diagnosis (**c**) in ESCC patients with high and low relative PTK7 expression. Means and 95% confidence intervals are represented as solid and dotted lines, respectively. Numbers of patients are shown in parentheses.

The associations between *PTK7* mRNA levels and relative risk and survival were assessed from 93 ESCC samples in the database from TCGA; three outliers were removed (see Supplementary Fig. S3) using the LPEseq tool^30^. Our analysis using a Cox proportional hazards model showed that the relative risk in ESCC patients decreased with increasing *PTK7* mRNA levels (Fig. 5a, left). As *PTK7* mRNA levels are already high in tumor tissues of ESCC patients, we were not able to detect a change in risk with lower levels of expression. However, relative risk was not associated with *PTK7* expression in EAC patients (Fig. 5a, right).

To further explore how *PTK7* mRNA levels affect ESCC patients, we categorized ESCC samples as either “PTK7 high” (*n* = 62) or 31 “PTK7 low” (*n* = 31) using a log-rank test of overall survival. There were no significant associations between relative PTK7 expression and clinical parameters, such as age at diagnosis, sex, pathologic stage, neoplasm histologic grade, and lymph node metastasis (Table 1). However, a Kaplan–Meier survival plot showed that ESCC patients in the PTK7-high group had a significantly longer overall survival than those in the PTK7-low group (*P* = 0.011) (Fig. 5b). We also compared the relative risk between PTK7-high and PTK7-low groups with regard to the age at diagnosis in a spline survival analysis. As in other cancers, the relative risk increased with an increase in age at diagnosis in both groups of patients with ESCC. Interestingly, the PTK7*-*high group showed a lower relative risk than the PTK7-low group over the range of ages analyzed (*P* = 0.024) (Fig. 5c).

**Table 1:** Association analysis between PTK7 expression and clinicopathological features of 93 ESCC patients.

Clinicopathological features	Characteristic	PTK7 expression		*P*-value
		Low (n = 31)	High (n = 62)	
Age at diagnosis	> = 57	18	34	0.83
	<57	13	28	
Sex	Female	5	9	1.00
	Male	26	53	
Pathologic stage	I	3	4	0.79
	II	18	36	
	III	8	19	
	IV	2	2	
	NA	0	1	
Neoplasm histologic grade	G1	6	10	0.38
	G2	15	32	
	G3	5	16	
	GX	5	4	
Lymph nodeMetastasis Radiographic evidence	Yes	10	10	0.59
	No	17	25	

## Discussion

We analyzed samples comprising a broad range of cancer types in the database from TCGA and found that *PTK7* mRNA levels are high in many cancers, including endometrial and esophageal cancers. A further examination in esophageal cancers revealed that *PTK7* mRNA level is higher in cancer tissues than that in adjacent normal tissues, and the level is higher in ESCC than that in EAC. Thus, we hypothesized that PTK7 plays a role in tumorigenesis of ESCC.

The oncogenic role of PTK7 has been reported in various cancer cells including ESCC cells. PTK7 knockdown decreased proliferation, survival, wound healing and invasion by inhibiting ERK, JNK, p38, Akt and FAK activation in ESCC TE-10 and TE-11 cells^4^. Overexpression of PTK7 induced cell proliferation and invasion and suppressed apoptosis in ESCC TE-5 and TE-9 cells^24^. Consistently, we found here that overexpression of PTK7 increased proliferation in PTK7-low ESCC TE-5 and TE-14 cells and that PTK7 knockdown decreased it in PTK7-high ESCC TE-6 and TE-10 cells. However, overexpression of PTK7 decreased proliferation in PTK7-high ESCC TE-6 and TE-10 cells, suggesting that the PTK7 expression level does not correlate linearly with the oncogenic potential. To further understand the relationship between the level and the oncogenic potential of PTK7, PTK7 expression level was modulated by transfection of a PTK7 knockdown vector or a PTK7 expression vector. As PTK7 expression levels increased, the proliferation, migration, and invasion of ESCC cells were enhanced. However, when PTK7 expression levels exceeded an optimal level, these characteristics were suppressed. Thus, the oncogenic properties of ESCC cells are biphasically regulated with increasing PTK7 expression levels. In accordance with the oncogenic effects of PTK7 expression, we also demonstrated that tyrosine phosphorylation of cellular proteins and phosphorylation of Src, Akt, and ERK was also biphasically modulated with regard to PTK7 expression in ESCC cells.

Our findings for a biphasic regulation of oncogenicity by PTK7 in ESCC cells correspond with those of our previous study showing a biphasic regulation of angiogenesis by PTK7. In that study, the VEGF-induced phosphorylation of KDR and tube-formation in human umbilical vein endothelial cells (HUVECs) and *in vivo* angiogenesis initially increased and then decreased with increasing PTK7 expression levels^25^. We showed that PTK7 interacts with KDR but not with FLT-1 in HUVECs, even though KDR and FLT-1 are both VEGF receptors. It appears that when levels of PTK7 are lower than that of KDR, PTK7 helps KDR molecules to oligomerize and activate, whereas high amounts of PTK7 inhibit this. As shown in the analysis of the functional mechanism of PTK7 in angiogenesis, we hypothesized that PTK7 would bind to some RPTKs in ESCC cells. However, KDR, which binds to PTK7 in endothelial cells, is not expressed in ESCC cells (data not shown). Further studies are needed to determine which binding partners such as catalytically active RPTKs are responsible for these effects in ESCC cells.

An analysis of the patient data provided in the database from TCGA revealed that the overall survival of ESCC patients was higher in those with higher PTK7 levels. Of note, the relative expression of PTK7 in ESCC is comparable to that in lung cancer samples (Fig. 1a and c), and PTK7 overexpression was also shown to decrease oncogenic effects in lung squamous cell carcinoma cells^29^. Thus, in some cancer types with comparable, or even higher, PTK7 expression, the suppressive effect of PTK7 on oncogenic characteristics may be apparent.

The inhibition of PTK7 has been suggested as a novel therapeutic target for various cancers such as colorectal cancer and atypical teratoid rhabdoid tumors^31,32^, and some anticancer agents under development involve PTK7-targeted antibody-drug conjugates^33^ or PTK7 aptamers^34^. However, caution should be used with this strategy when considering the evidence showing PTK7 can biphasically regulate tumorigenesis, as the inhibition of PTK7 activity or removal of PTK7-positive cells may induce tumor progression. Therefore, PTK7-targeted therapy should be applied to only for cancers with either low PTK7 expression or where the inhibition of PTK7 has been shown to reduce tumorigenicity.

Taken together, our data show that tumorigenesis in ESCC cells initially increases and then decreases with increasing expression levels of PTK7 and that lower PTK7 expression in ESCC tissues is associated with a poorer prognosis. Therefore, a better understanding of the biphasic regulation of tumorigenesis by PTK7 will provide important information for the development of therapeutic means to control ESCC and other cancers that express PTK7.

## Materials and Methods

### Analysis of public dataset

A preprocessed RNA-seq count matrix and the associated clinical features of 10,048 samples, including 198 esophageal cancer samples were downloaded using *recount2*^35^ from TCGA (https://cancergenome.nih.gov/). To compare the *PTK7* mRNA levels among cancer tissue types, a scaling (library size) normalization was first conducted using LPEseq.^30^. Then log-transformed normalized mRNA levels for the 20 different cancer tissues were used for further analyses.

### Cell culture

Human ESCC TE-5, TE-6, TE-10, and TE-14 cells (RIKEN BioResource Center, Tsukuba, Japan) were maintained in Dulbecco’s modified Eagle’s medium (DMEM; Gibco of Thermo Fisher Scientific) supplemented with 10% fetal bovine serum (FBS), 100 U/ml penicillin, and 100 μg/ml streptomycin at 37 °C in 5% CO_2_.

### Western blotting and antibodies

Western blotting was performed as described previously^25^. The anti-PTK7 antibody was described previously^36^. Antibodies against phospho-ERK, ERK2, and β-actin were purchased from Santa Cruz Biotechnology (Santa Cruz, Dallas, TX, USA). Antibodies against phospho-Src (Tyr416), Src, phospho-Akt (Ser473), and Akt were purchased from Cell Signaling Technology (Danvers, MA, USA). The anti-phospho-tyrosine antibody (clone 4G10) was purchased from Millipore (Billerica, MA, USA). Horseradish peroxidase-conjugated goat anti-mouse IgG and goat anti-rabbit IgG were purchased from KOMA Biotech (Seoul, Korea).

### Expression and knockdown vectors

An expression vector for human PTK7 with a C-terminal Flag tag (pcDNA3-PTK7-FLAG) and a knockdown vector for human PTK7 (pLKO.1-shRNA-PTK7-6434) and an empty knockdown vector (pLKO.1-control) (Sigma-Aldrich, St. Louis, MO, USA) were described previously^4,25^.

### Transfections

Subconfluent cells were transfected using Lipofectamine 2000 (Invitrogen, Carlsbad, CA, USA) according to the manufacturer’s instructions. Cells were harvested for analysis 24 or 48 h after transfection.

### Cell proliferation assay

Cells (0.1 ml, 3 × 10^4^ cells/ml) were plated in 96-well plates and maintained in DMEM supplemented with 5% FBS for up to 3 days. Cell proliferation was measured with the colorimetric MTT assay as described previously^4^.

### Chemotactic migration and invasion assays

The chemotactic migration assay was performed as described previously^36^. Briefly, detached cells (1 × 10^5^ cells/0.2 ml) were placed on a Transwell filter of which the bottom surface had been coated with 10 μl of 0.1% gelatin. The lower compartment of the well below the filter was filled with 0.6 ml DMEM with 5% FBS as the chemoattractant. After a 24-h incubation at 37 °C, cells that had migrated to the bottom surface of the filter were fixed with 3.7% formaldehyde for 15 min and stained with 5% crystal violet for 5 min. Invasion assays were identical to the chemotactic migration assay, except that the top surface of the filter was coated with 25 μg of growth factor-reduced Matrigel and dried overnight. After the removal of non-migrated or invaded cells with a cotton swab, the stained cells were solubilized with 1% SDS and the absorbance was measured at 600 nm.

### Statistical analysis

Statistical analyses were performed using R (http://www.r-project.org) and Excel (Microsoft, Redmond, WA, USA) software. For group comparisons, Student’s *t-*tests were performed to analyze the differences in *PTK7* mRNA levels between esophageal cancer samples and adjacent normal tissue samples and to assess the proliferation, migration, and invasion of ESCC cells transfected with various amounts of PTK7 expression vectors. A Fisher’s exact test was conducted to provide the significance of the association between clinicopathologic features and *PTK7* mRNA levels. A Kaplan–Meier plot and its corresponding *P* value from a log-rank test were used to provide the significance of survival between PTK7-high and -low groups. Cox proportional hazards regressions including nonlinearity of PTK7 expression and age at diagnosis were performed using “survival” and “splines” R packages to estimate the relative risk associated with *PTK7* mRNA level and age at diagnosis. For all tests, *P* values less than 0.05 were considered statistically significant.

## Electronic supplementary material

Supplementary Information
