# Supplementary material for: Biphasic regulation of tumorigenesis by PTK7 expression level in esophageal squamous cell carcinoma
Source: Sci Rep. 2018 Jun 4;8:8519. doi: 10.1038/s41598-018-26957-6 (PMC5986804; doi:10.1038/s41598-018-26957-6)
Supplement: Supplementary file 1 — Supplementary Information [file 41598_2018_26957_MOESM1_ESM.pdf]

Supplementary Information

Biphasic regulation of tumorigenesis by PTK7 expression level in esophageal squamous cell carcinoma

Won-Sik Shin[1], Jungsoo Gim[2], Sungho Won[3,4], and Seung-Taek Lee[1]*

[1]Department of Biochemistry, College of Life Science and Biotechnology, Yonsei University, Seoul, Republic of Korea; [2]Department of Biomedical Science, College of Natural Science, Chosun University, Gwangju, Republic of Korea; [3]Graduate School of Public Health, Seoul National University, Seoul, Republic of Korea; [4]Interdisciplinary Program for Bioinformatics, Seoul National University, Seoul, Republic of Korea

***Corresponding author:**

Seung-Taek Lee, Department of Biochemistry, College of Life Science and Biotechnology, Yonsei University, Seoul 03722, Republic of Korea. Phone: +82-2-2123-2703, FAX: +82-2-362-9897, E-mail: stlee@yonsei.ac.kr

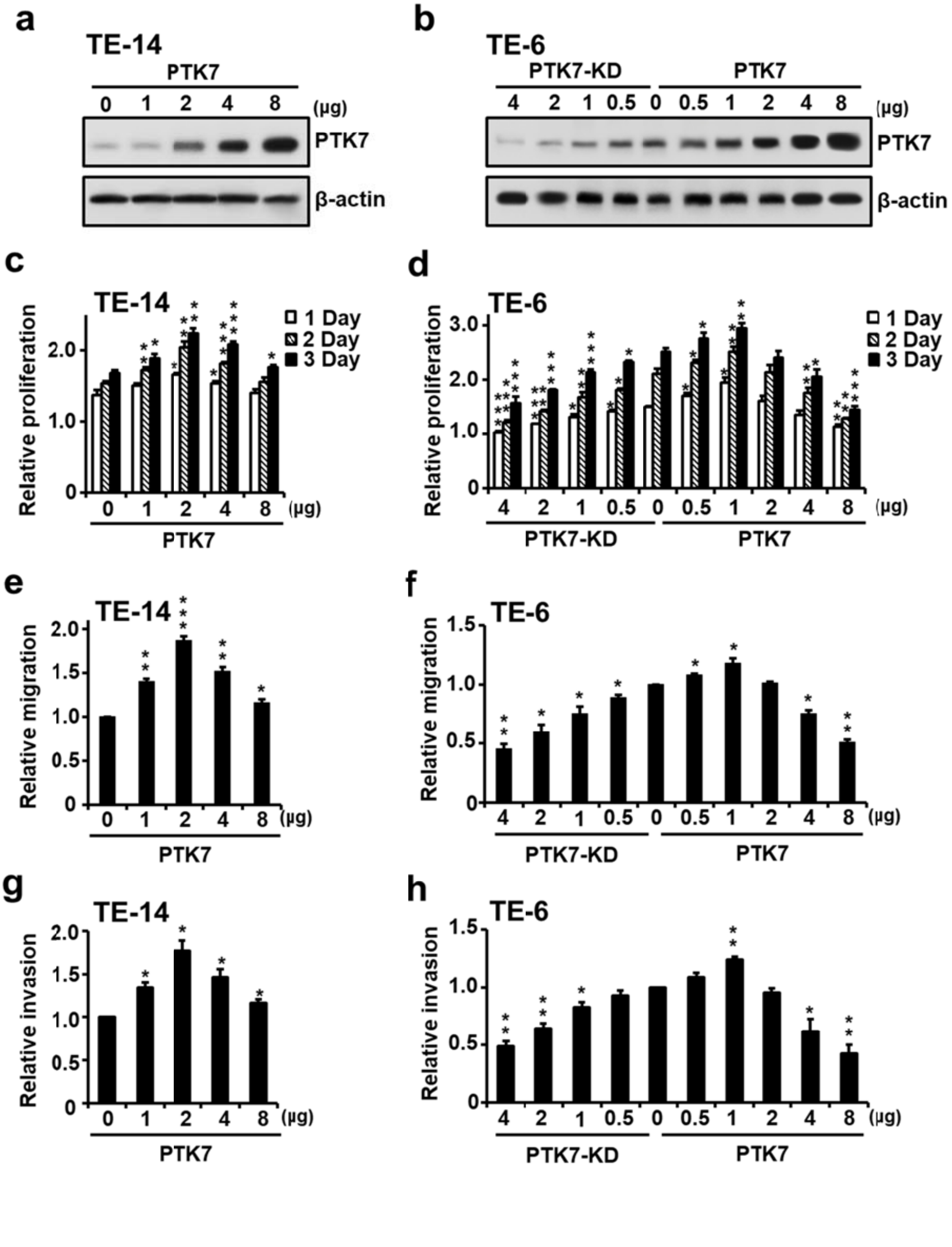
a
TE-14
PTK7
0 1 2 4 8 (μg)
PTK7
β-actin
b
TE-6
PTK7-KD
PTK7
4 2 1 0.5 0 0.5 1 2 4 8 (μg)
PTK7
β-actin
c
TE-14
Relative proliferation
1 Day
2 Day
3 Day
0 1 2 4 8 (μg)
PTK7
d
TE-6
Relative proliferation
1 Day
2 Day
3 Day
4 2 1 0.5 0 0.5 1 2 4 8 (μg)
PTK7-KD
PTK7
e
TE-14
Relative migration
0 1 2 4 8 (μg)
PTK7
f
TE-6
Relative migration
4 2 1 0.5 0 0.5 1 2 4 8 (μg)
PTK7-KD
PTK7
g
TE-14
Relative invasion
0 1 2 4 8 (μg)
PTK7
h
TE-6
Relative invasion
4 2 1 0.5 0 0.5 1 2 4 8 (μg)
PTK7-KD
PTK7

Supplementary Figure S1. Biphasic oncogenic regulation of ESCC TE-6 and TE-14 cells by PTK7 expression. Representative Western blots showing PTK7 levels in PTK7-low TE-14 cells 24 h after transfection with increasing amounts of the PTK7 expression vector (pcDNA3-PTK7-FLAG; PTK7) (a), and in PTK7-high TE-6 cells transfected with various amounts of a PTK7 knockdown vector (pLKO.1-shRNA-PTK7-6434; PTK7-KD) or the PTK7 expression vector (b). Proliferation of the TE-14 (c) and TE-6 (d) cells was analyzed for 3 days. Migration and invasion of the TE-14 (e and g) and TE-6 (f and h) cells were analyzed 24 h after transfection. Each bar represents the mean ± standard deviation from three independent experiments. *$P < 0.05$, **$P < 0.01$, ***$P < 0.001$ *vs.* day 0 (c and d) or 0 μg of PTK7 expression vector (e to h). Samples derived from the same experiment and gels/blots were processed in parallel. The blots were cropped to focus upon the specific proteins indicated. Uncropped images of blots are shown in Supplementary Fig. S4.

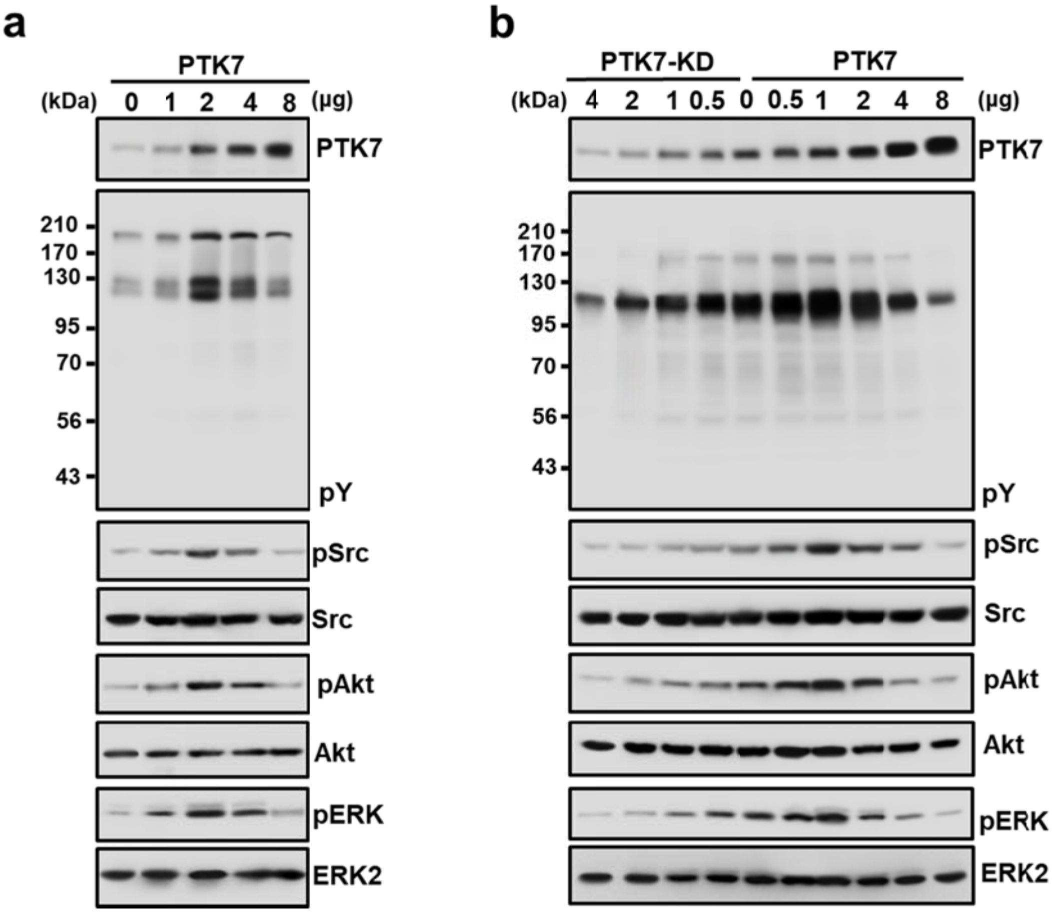

Supplementary Figure S2. Biphasic regulation of protein phosphorylation in ESCC TE-6 and TE-14 cells by PTK7 expression. Representative Western blots from PTK7-low TE-14 cells 48 h after transfections with increasing amounts of the PTK7 expression vector (pcDNA3-PTK7-FLAG; PTK7) (a), and PTK7-high TE-6 cells transfected with various amounts of a PTK7 knockdown vector (pLKO.1-shRNA-PTK7-6434; PTK7-KD) or the PTK7 expression vector (b). Levels of tyrosine-phosphorylated cellular proteins (pY), as well as phosphorylated Src, Akt, and ERK, are shown. Numbers to the left of the blots indicate the molecular mass of the marker proteins (kDa). Samples derived from the same experiment and gels/blots were processed in parallel. The blots were cropped to focus upon the specific proteins indicated. Uncropped images of blots are shown in Supplementary Fig. S4.

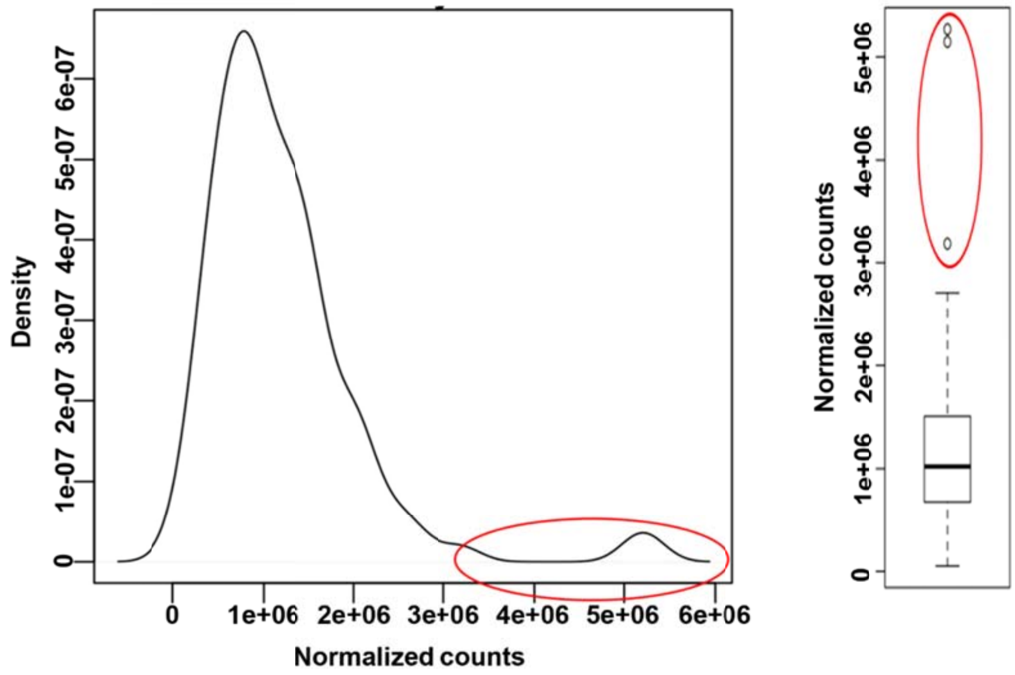

Supplementary Figure S3. *PTK7* mRNA expression outliers in ESCC samples in the dataset from TCGA. *PTK7* mRNA levels in ESCC samples from the dataset are shown in a density plot and a boxplot with the original scales. Three outliers were identified (red circles).

Fig. 2a

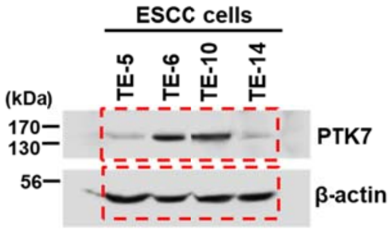

Fig. 2b

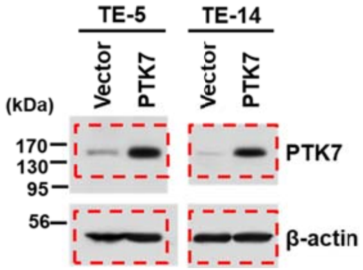

Fig. 2c

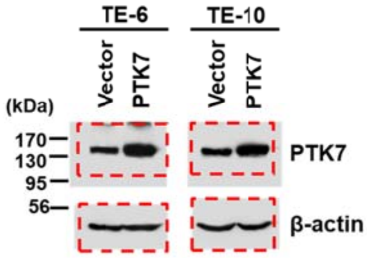

Fig. 3a

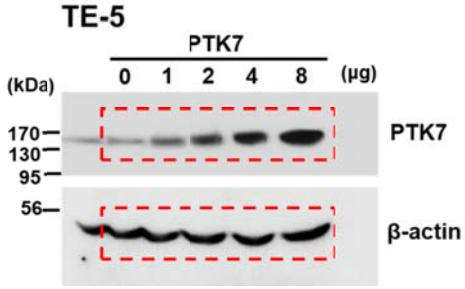

Fig. 3b

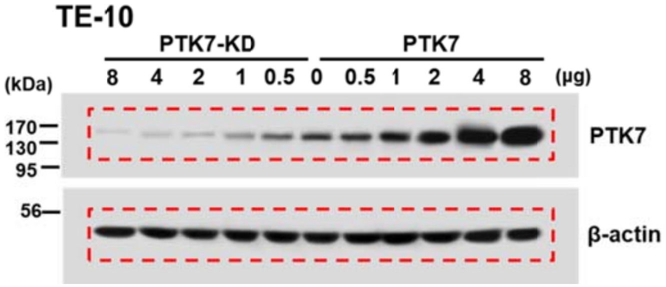

Fig. 4a

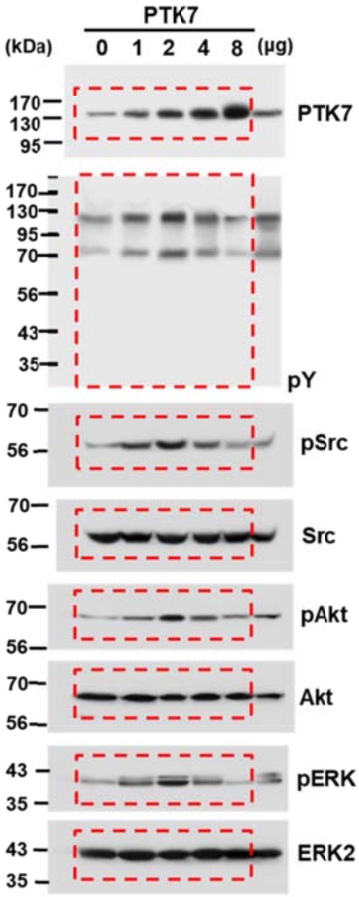

Fig. 4b

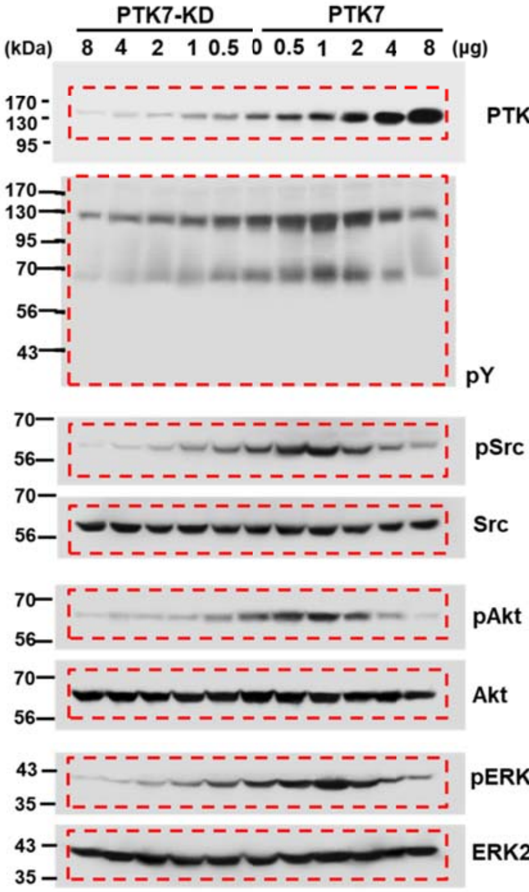

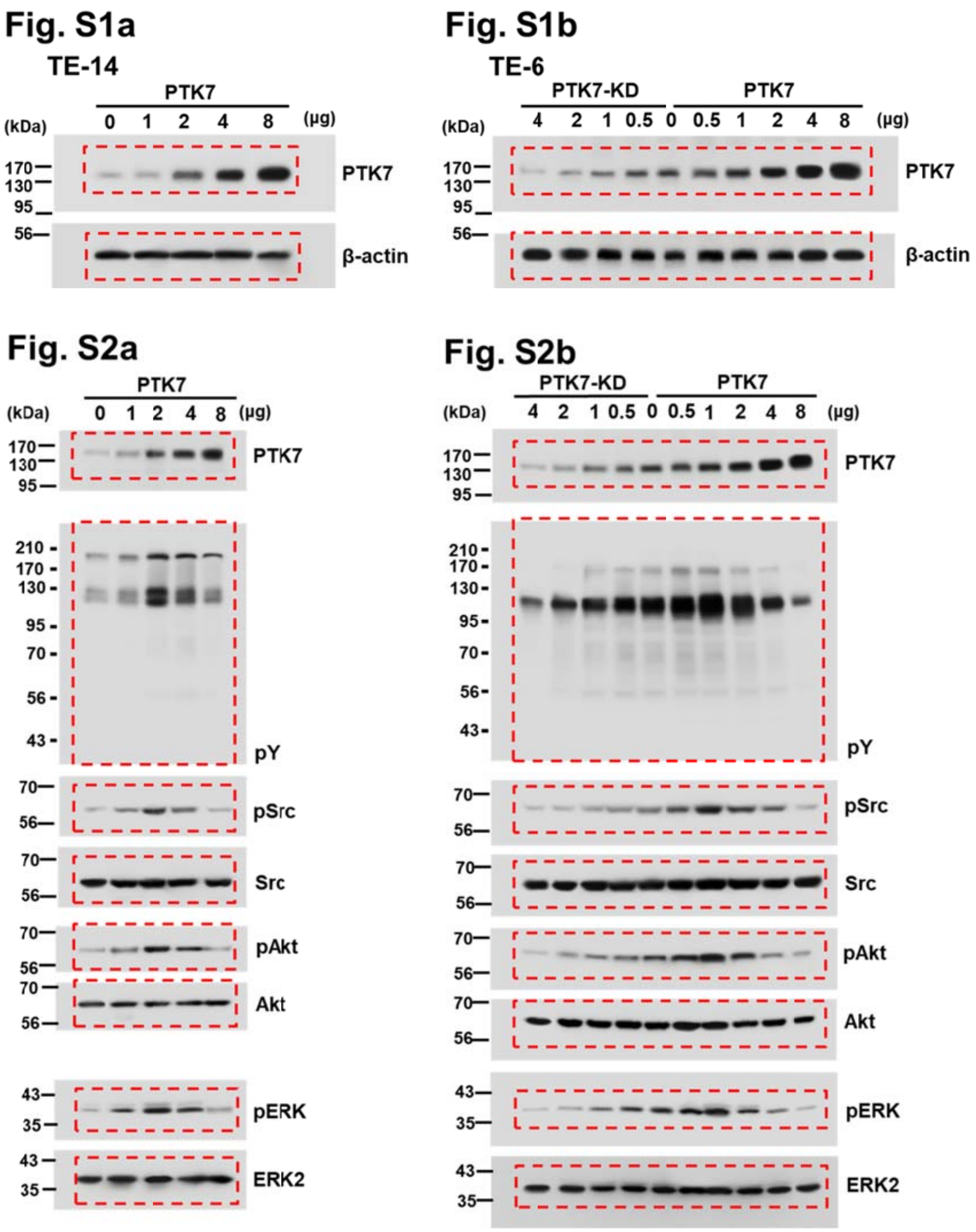

Supplementary Figure S4. Uncropped scans of blots and gels displayed in the main and supplementary figures
